# A Worksite Health Education Workshop as Empowerment Intervention for Health Promotion in the National Research Centre of Egypt

**DOI:** 10.3889/oamjms.2016.093

**Published:** 2016-08-28

**Authors:** Nagat Mohamed Amer, Zeinab M. Monir, Mai Sabry Saleh, Heba Mahdy-Abdallah, Salwa Farouk Hafez

**Affiliations:** 1 *Environmental Research Division, Environmental and Occupational Medicine Department, National Research Centre, Cairo, Egypt*; 2 *Medical Research Division, Department of Child Health, National Research Centre, Cairo, Egypt*

**Keywords:** Occupational stress, health education, health promotion, empowerment, workshop

## Abstract

**AIM:**

The study aimed to assess worksite health education workshops as a successful tool for health promotion of employees.

**MATERIAL AND METHODS:**

A one day workshop was held for individuals engaged in research activities in the National research Centre of Egypt at the worksite. Its main objective was to highlight the nature, causes, symptoms and management of job stress. Participants were asked to fill a personality assessment sheet, a self-reported questionnaire for job satisfaction. Other questionnaires for assessment of falsification of type and some socio-demographic data were filled by the attendants. A concise survey was introduced at the end of the workshop for feedback collection.

**RESULTS:**

Attendants of the workshop were 36 subjects mainly females (94.4%). Mean age was 40.5 years with 63.9% of participants at their postdoctoral studies stage. Participants were at midway in the scale of job satisfaction (3.3) and did not suffer from falsification (0.3). The feedback survey score (11.5) showed great acceptance for the intervention. Special interest in the topic of stress was reported by 35.1% of attendants who found it the best item in the workshop and the interactive manipulation came next as declared by 18.9% of the participants.

**CONCLUSION:**

Worksite health education workshops seem to be a successful practice for empowerment in the Egyptian workplace.

## Introduction

Empowerment is a process by which people, organisations and communities gain mastery over their affairs [1]. Individual empowerment -in particular- which is also known as psychological empowerment, focuses more on people feeling and actually having a sense of control over their lives. As reported, the ability to create such ’sense of control’ could show a direct effect on improving an individual’s mental and physical health [2]. Moreover, evidence from research ensures the effectiveness of empowerment interventions –in general- in their ability to produce improved health impacts [3, 4].

In the context of the conceptual theory and applications, ’empowerment’ is closely related to ’health promotion’. Health promotion is defined as the process of enabling people to increase control over, and to improve, their health [5]. Health education represents one discipline of ’health promotion’ [6] which include educational efforts to influence lifestyles that guard against ill-health [7].

According to the WHO [8] people in developing countries are subjected to increasing work-related stress mainly due to globalisation and changes in the nature of work. For that reason, the WHO [8,9] encourages interventions that improve awareness with hazards of stress and promote suitable ways of coping with demanding working conditions. On practical basis, a variety of health deficits caused by stress, especially psychological ones were found to be improved by enhancing self-awareness and self-efficacy as well as changing the irrational thoughts as part of health education programs [10].

In consequence, we aimed to introduce a workshop for health education against workplace stress for the specific category of workers: scientific researchers as an empowerment intervention for health promotion. Feedback was collected after the intervention for evaluation of the process and assessment of the response of the study group.

## Methodology

A health educative *one day workshop* with title: “Let your job be your friend” was held by the Principal Investigator, Co-Investigator and some of the team members of the internal project funded by the National Research center (NRC) of Egypt entitled: “Increasing productivity of young researchers in NRC through learning style preference assessment and investigation of some related professional factors”. The workshop was held in the main auditorium of the NRC. Attendants were asked to fill a survey at the end of the workshop to record their feedback. Ethical committee approval was taken in advance.

### Participants

Attendants of the workshop were 36 subjects (2 males and 34 females) working at the different departments and divisions of the NRC. All participants completed the whole workshop and showed a high level of interaction.

### Description of the workshop

The workshop first explained its objectives and presented a short scientific background to the audience as well as some instructions to help them obtaining maximum benefit from the intervention introduced.

Topics of the workshop encompassed an introduction to workplace stress and its hazardous effect on health, hormones involved in stress induction, stress management and coping strategies and finally subjective stressors according to personality traits and hazardous effect of falsification of the type in the work environment. Interactive manipulation of the various topics was the main feature of the intervention.

### Measures and scales

Participants in the workshop were asked to fill a personality assessment sheet modelled after the Myers-Briggs Type Indicator (MBTI) and guided by Cognitive Style Inventory [11]. The questionnaire investigated the 16 different personalities of the MBTI where each personality is represented by four letters representing the four famous dichotomies: (Extrovert (E)/Introvert (I), Sensing (S)/Intuition (N), Thinking (T)/Feeling (F) and Judging (J)/Perceiving (P).

The self-reported questionnaire for job satisfaction designed by Andrews and Withey [12] was used in the workshop to assess the degree to which attendants liked their jobs. It consisted of five questions asking about the different aspects featuring an individual’s working environment (job, co-workers, work itself, the amount of work and facilities provided). A Likert scale ranging from one (delighted) to seven (terrible) described their degree of satisfaction with work. The average score was then calculated to measure overall state of job satisfaction ranging from 1 indicating the state of delight and 7 indicating feeling terrible.

Another questionnaire was presented to participants for identification of falsification of type inside the work environment [13]. The questionnaire consisted of 14 statements describing one’s feelings and behaviours towards his workplace. Participants were asked to respond to all the sentences with ’Agree’ (scores for 1), ’don’t agree’ (scores for zero) or ’sometimes yes and sometimes no’ (scores for half). The total score was calculated by summing up all responses and calculating the average value. The scale of falsification ranged from zero to 1 where zero represents the absence of falsification and 1 mean complete falsification of type at work.

Double translation-retranslation and face validation were done for all the tools in use. Participants were asked to score the different questionnaires to assess themselves and participate in the workshop. Demographic data were finally collected which included age, gender, educational level (academic degree), address district (near or far from work), residence, monthly income, position and if someone suffers from any chronic disease (hypertension, diabetes …).

### Feedback collection

For the purpose of feedback collection, a short survey was designed up of five forced choice questions with allowed answers yes, no or don’t know and three open questions in which participants were asked to mention the best thing in the workshop, to give their own recommendations, and to add a positive and/or negative comments if present.

The forced choice questions were:

Q1. If this workshop is held again I will encourage my friends to attend it.
Q2. If this workshop is extended for more than one day I will try to attend it.
Q3. There are many workshops speaking about the same topic of this workshop.
Q4. The topic of the workshop is new for me.
Q5. This workshop manipulates its topic in an innovative manner.

Calculating the number of points for each forced choice question was as follows: Yes accounted for two points and, one point for don’t know, and zero for the answer (no), except the third question, were, answering with no gets 2 points and zero for yes. Two points were added to the score for each positive comment, and 2 points were subtracted corresponding to each negative comment.

### Statistical analysis

Statistical analysis was carried out using the statistical package for social sciences, version 16 for windows (SPSS Inc., USA). Descriptive statistics including frequency distribution, mean and standard deviation: to describe different characteristics and the studied scores. One sample t-test was used to test whether the average of the sample differs significantly from a population mean. Two groups comparison was done using Independent sample t-test. Pearson Correlation (r) was used to test the association between two variables. A P value less than 0.05 was considered as statistically significant.

## Results

As shown in Table 1, total participants in the workshop reached 36 from different divisions in the NRC. Research members from the Environmental Research division represented the highest contribution in the workshop (19.4%) followed by the division of Food Industry and Nutrition, Pharmaceutical Industries division and Genetic Engineering and Biotechnology division with the percentage of 11.1 % each.

**Table 1:** Socio-demographic, some work stress related factors and personality types in the studied group

		Number	Percentage %
Gender	Male	2	5.6
	Female	34	94.4
Divisions	Agriculture and Biology	3	8.3
	Chemical Industries	3	8.3
	Engineering	2	5.6
	Environmental Sciences	8	22.2
	Food Industry and Nutrition	4	11.1
	Genetic Engineering and Biotechnology	4	11.1
	Inorganic Chemical Industries and Mineral Resources	3	8.3
	Medical Sciences	1	2.8
	Pharmaceutical Industries	4	11.1
	Physics	1	2.8
	Textile Industries	1	2.8
	Veterinary	1	2.8
	Human Genetics & Genome	1	2.8
Income	<3000 LE	2	5.6
	<5000 LE	9	25
	>5000 LE	25	69.4
Highest Degree	Bachelor	9	25.0
	Master of Science	4	11.1
	Medical or Philosophy Degree	23	63.9
Position	Specialist	6	16.7
	Assistant Researcher	8	22.2
	Researcher	11	30.6
	Assistant Professor	6	16.7
	Professor	5	13.8
Residence	Urban	34	94.4
	Rural	2	5.6
Place of Work	Near from home	16	44.4
	Far from home	20	55.6
Chronic Diseases	Present	14	38.9
	Absent	22	61.1
Personality Type	ISTJ	8	22.2
	ISFJ	3	8.3
	INFP	1	2.8
	INTJ	1	2.8
	INFJ	1	2.8
	ISFP	1	2.8
	INTP	1	2.8
	ESTP	1	2.8
	ESTJ	3	8.3
	ESFJ	11	30.6
	ENFJ	3	8.3
	ESFP	2	5.6

The average age of years for the included staff was 40.5 ± 11.5, ranging between 24 and 62 years. Females (n = 34) highly exceeded males (n = 2) with percentages 94.4% and 5.6%, respectively which obeys normal distribution of researchers working in the NRC. Monthly income for most of the sample (70.6%) exceeded 5000 Egyptian pounds with 63.9% of the population sample holding Medical Degree (MD) or Doctoral Degree of Philosophy (PhD). Participants were nearly equally distributed over the different positions in the NRC with the slight increase among researchers (31.4%). Urban residence (97.1%) greatly predominated rural residence (2.9%) and 40% of the sample showed to suffer from chronic diseases. As for personality types, not all the 16 MBTI types were represented by the sample but only 12 of them as shown in Table 1.

The ESFJ personality predominated (30.6%) followed by the ISTJ (22.%) while the INFP, INTJ, INFJ, ISFP, INTP and ESTP were the least represented (2.8% each). The mean score of falsification of the type for the workshop participants as shown in Table 2 reached 0.3 (SD = 0.19) with a good indication of the nearly absence of falsification within the sample.

**Table 2:** Falsification of type, job satisfaction and feedback survey scores of participants

	Minimum	Maximum	Mean ± SD
Falsification of Type	0.00	0.78	0.3 ± 0.19
Job Satisfaction	0.24	4.60	3.3 ± 0.85
Feedback Survey Scores	6	14	11.5 ± 2.06

As for job satisfaction, the mean score showed a general state of satisfaction with work (3.3). At the same time, the survey showed a mean score of 11.5 which indicates a high percentage of acceptance (82%) for the workshop which is described in details in tables (3) and (4). Data in Table 3 describes frequencies of responses to force choice questions of the survey with yes, no or don’t know. As shown, the mean percentage of positive responses (’yes’ for all questions except the third which takes ’no’) is 82.2%.

**Table 3:** Frequency table for responses to force choice items of the feedback survey with Yes, No or Don’t know

Serial	Items	Number of respondents	YES No. (%)	NO No. (%)	Don’t know No. (%)
1	If this workshop is held again I will encourage my friends to attend it.	35	35 (100%)	0	0

2	If this workshop is extended for more than one day I will try to attend it.	35	30 (85.7%)	1 (2.9%)	4 (11.4%)

3	There are many workshops speaking about the same topic of this workshop.	35	1 (2.9%)	17 (48.6%)	17 (48.6%)

4	The topic of the workshop is new for me.	34	30 (88.2%)	3 (8.8%)	1 (2.9%)

5	This workshop manipulates its topic in an innovative manner.	35	31 (88.6%)	1 (2.9%)	3 (8.6%)

Positive comments (42.9%) as represented in Table 4 highly exceeded negative comments (11.1%) where the former included an appraisal of the topic (new, interesting and important) primarily and its manipulation (simplified, interactive and interesting) secondarily. Recommendations were offered by 51% of participants and encompassed more illustrations, follow-up, extended time for the workshop, copy of the material content and more active participation from attendants while 94.1% stated their opinion on the best thing in the workshop as detailed in Table 4.

**Table 4:** Descriptive data showing responses of participants to open-ended questions in the feedback survey

Survey Items		Number	Percentage %
Best item in the workshop		33	89.1
	The main topic (interesting, novel, new information)	13	35.1
	Manipulation of the topic	7	18.9
	Stress management	5	13.5
	Personality topic	3	8.1
	Performance and skills of trainers	5	13.5
Recommendations		18	51.4
	More illustrations	3	8.3
	Follow-up	3	8.3
	Extend the duration of the workshop	6	16.7
	Provide copy of the material content	5	13.5
	More active participation from attendants	1	2.8
Positive Comments		15	42.9
Negative Comments		4	11.1

The response to the workshop according to personality differences between participants through comparison of mean scores of the survey is shown in Table 5. The Feeling have shown to be significantly more responsive and convinced by the workshop than the Thinking type

**Table 5:** Comparing means for feedback survey scores between participants with different personality traits

MBTI Dichotomies		N	Mean ± SD	p value
Orientation to World	Introverted	16	10.81 ± 2.37	0.09
	Extroverted	19	12.00 ± 1.63	
Take in Information	Sensing	28	11.64 ± 1.66	0.29
	Intuitive	7	10.71 ± 3.30	
Make Decisions	Thinking	14	10.50 ± 2.59	0.02*
	Feeling	21	12.10 ± 1.34	
Adapt to the World	Judging	29	11.62 ± 1.86	0.31
	Perceiving	6	10.67 ± 2.94

* Significance at p-value ≤ 0.05.

As for the study of Pearson correlation, a negative correlation was found between falsification of type and both income (p = 0.039) and degree of job satisfaction (p = 0.002), which means that falsification increases in case of low income and decreased job satisfaction.

Upon comparing means of scores of falsification of the type using student t-test, it showed significantly to have a lower mean (0.25 ± 0.2) with (p = 0.041) for a participant who are MD or PhD holders compared to those who have only a bachelor or master degree (0.4 ± 0.16).

## Discussion

The job represents one of the major stressors for many people [14]. According to NIOSH [15], job stress is more strongly associated with health complaints than financial or family problems. Work-related stress in developing countries –in particular- is even made worse by a broad spectrum of factors outside the work environment [8]. Identifying stressors and learning how to face them crucially help in reducing distress in people lives [16]. As noted by the Canadian Mental Health association [17], employees lack the ability to recognise signs and symptoms of deteriorating workplace stress which subject them to serious physiological and psychological conditions in addition to the negative impact that could be exerted on the workplace productivity and profit. For that reason, we tried to implement our workshop for employees’ empowerment which we consider as *public health intervention* that intends to protect the psychological health and prevent illness [18].

Despite the great diversity of empowerment practices at the workplace [19], we preferred for our intervention to be a one-day health education workshop at the worksite since worksite interventions proved to have the capacity to reach a big proportion of the working population [14]. Moreover, health education is highly encouraged in particular as it represents an important part of the health promotion activities currently occurring in the WHO Eastern Mediterranean Region countries including Egypt [7].

A survey was introduced to participants at the end of the workshop for feedback collection as a method for evaluation of the process as it is difficult to accurately determine the impact on the individual health outcomes later on [4]. The survey was designed in light of the definition of evaluation which regards it as “the systematic examination and assessment of features of an initiative and its effects, in order to produce information that can be used by those who have an interest in its improvement or effectiveness” [20].

According to the presented evaluation, we found a great degree of acceptance regarding the intervention introduced. It was represented by the high mean values (11.5 ± 2.1) of the survey score that indicates the positive opinion of participants, 88.2% were interested in the topic and 88.6% found the manipulation of the material interesting and innovative. Moreover, 100% of the participant declared their willingness to re-attend the intervention if repeated. A percentage of 85.7 of them preferred that the workshop could have been extended for a longer period. These results ensure a great deal of serious need for such kind of interventions and highlight the importance of paying attention to the problem of work-stress in our community. As reported by the WHO [8] very little data is available from developing countries concerning the magnitude of causes and consequences of work-related stress. This could explain the urgent need for such kind of health education intervention as shown from our results.

As part of the process evaluation, we had to screen some of the individual characteristics of participants who received our intervention. Personality, age, gender, level of education, and family situation are reported to influence an individual’s ability to cope with work demands and may also interact with risk factors at work and either exacerbate or buffer their effects [8]. Another main interest in such screening was to test job satisfaction and the prevalence of stress according to falsification of type together with some work-related factors which might influence them. According to statistical analysis although the sample showed good job satisfaction (3.3) and low value on the scale of falsification (0.3), yet significant difference was detected that revealed the presence of some kind of stress exerted on researchers who haven’t yet received their PhD/MD (0.4) compared to PhD/MD holders (0.25) at p < 0.05. Falsification of type -which reflects signs and symptoms of stress in response to not convenient working conditions- also showed significant negative correlation at p < 0.05 with low income among researchers. Researchers in this stage (specialists and assistant researchers) are mostly subjected to some or all of the following factors which are highly reported to be sources of work stress: low income, high job demands, time pressure, a lack of control over workload and work processes, lack of social support from colleagues and/or supervisors (occasional), lack of participation in decision-making, job insecurity, lack of opportunity for growth, lack of advancement or promotion, irregular working hours and others.

As for the relation between personality type and response to the workshop, feeling style score in the survey significantly showed better satisfaction and convenience with the intervention than thinking style. This could be attributed to the nature of the thinking style that is not easily influenced and always tends to weigh pros and cons to judge things. No significant differences appeared among the rest of types which is a good sign of general acceptance. In this same context, one important point is the prominent role of individual differences among workers according to personality type in predicting whether certain job specifications will result in stress or not and specifying the kind of stressors that are the most irritating [8]. The workshop was able to clarify this point and introduced some tips that focus on the individual and promote ways of coping with demanding working conditions according to one’s nature. Participants were responsive with the idea and 8.1% chose such topic as the best thing in the workshop.

Recommendations as introduced by the attendants varied between the need of more illustrations, follow-up, extended time for the workshop, copy of the material content and more active participation from attendants. Negative comments were as follows: the need of more practical examples from life, time management, the need for more illustrations and the need of illustrative videos for personality types. As for positive comments they mainly stated both the topic and its manipulation were interesting and innovative.

Upon interpretation of the process evaluation results, we can clearly conclude a great deal of acceptance to such kind of interventions in our Egyptian community. Stress at work is proved to be an important topic and its management is a serious need. More developed programs with longer duration and variety of topics would be of crucial importance and are expected to receive a big flow of participants.
